# Health professionals’ readiness for and factors influencing electronic medical record systems implementation in Southern Oromia, Ethiopia, 2024: a cross-sectional study

**DOI:** 10.3389/fdgth.2025.1531315

**Published:** 2025-04-10

**Authors:** Miesa Gelchu, Geleta Chala, Gemechis Tuke, Gelgelo Wodessa, Angefa Ayele, Terefu Yambo, Anteneh Fikrie

**Affiliations:** ^1^ School of Public Health, Institute of Health, Bule Hora University, Bule Hora, Ethiopia; ^2^ West Guji Zonal Health Department, Oromia Health Bureau, Addis Ababa, Ethiopia; ^3^ Epidemiology and Biostatistics Department, School of Public Health, Institute of Health, Bule Hora University, Bule Hora, Ethiopia

**Keywords:** health professionals' readiness, electronic medical record system, West Guji zone, Borena Zone, Southern Oromia

## Abstract

**Background:**

The electronic medical record system is gradually being introduced in healthcare settings in high-income countries, but its adoption in low-resource settings like Ethiopia remains limited. There is a dearth of information regarding the readiness of health professionals to implement Electronic Medical Records system and the factors influencing this readiness, particularly in the study setting.

**Objective:**

The study aimed to evaluate the readiness of healthcare professionals in Southern Oromia for the implementation of the electronic medical record system.

**Methods:**

A facility-based cross-sectional study was conducted using self-administered questionnaires among 384 health professionals from May 1–30, 2024, at public hospitals in the Borena and West Guji zones in southern Ethiopia. Epi Data version 4.6 and SPSS version 27.0 were used for data entry and analysis, respectively. The study used multivariable logistic regression to analyse factors influencing health professionals’ readiness to implement electronic medical record systems, assessing adjusted odds ratios with a 95% confidence interval and a *p*-value below 0.05, which is considered to declare statistical significance.

**Results:**

Health professionals, 60.4% [95% CI: (55.5–65.3%)] expressed readiness to utilize the Electronic Medical Record system. Factors associated with electronic medical record system readiness included younger age [AOR = 2.66, 95% CI: (1.06–6.67)], personal computer ownership [AOR = 3.54, 95% CI: (1.76–7.11)], adequate computer skills [AOR = 2.49, 95% CI: (1.41–4.39)], high computer literacy [AOR = 2.67, 95% CI: (1.53–4.66)], knowledge of electronic medical record system [AOR = 2.56, 95% CI: (1.53–4.29)], and a favorable attitude towards electronic medical record system [AOR = 2.77, 95% CI: (1.66–4.63)].

**Conclusions:**

The study indicates that readiness for electronic medical record systems among health professionals is influenced by factors like younger age, computer ownership, skills, and positive attitudes. Interventions should target these factors, especially among older health professionals and those with limited digital literacy.

## Introduction

An electronic medical record system (EMRs) is a form of digital health technology used in healthcare organizations to collect, generate, and present health-related data to health professionals and facilitate information communication with authorized individuals (1, 2).

The national e-health strategy toolkit developed by the World Health Organization (WHO) and the International Telecommunication Union (ITU) defines EMR as “a computerized medical record used to capture, store, and share information among healthcare providers in an organization, supporting the delivery of health services to patients (3). Goal 3 of the United Nations Sustainable Development Goals (SDG) emphasizes the importance of data for decision-making in promoting good health and well-being (4). Additionally, Ethiopia's Second Health Sector Transformation Plan (HSTP II) proposes that “all of its citizens enjoy equitable and affordable access to all types of health services” as part of the Information Revolution transformation goal (5).

EMRs have the potential to address key challenges in healthcare systems, such as improving workflow, reducing medical errors, minimizing costs and treatment times, increasing revenue, and improving patient care by creating better linkages among caregivers (6). The use of electronic health tools enhances the monitoring of patient care, improves access to quality healthcare, and helps in managing global health risks (7, 8).

EMRs are gradually being implemented in healthcare settings in high-income countries. The use of EMRs has steadily increased over the last 15 years, with a recent global rise of 46% (9). Many studies have examined the adoption of EMRs in primary care settings in developed countries such as Canada, the United States, and the United Kingdom. These countries have advanced levels of EMRs adoption (10, 11). However, only 35% of lower-middle-income and 15% of low-income countries have nationally adopted electronic record systems in health institutions, which also vary across different settings (1).

Readiness encompasses various dimensions, including technical skills, organizational support, and the willingness to undergo significant behavioural changes associated with the adoption of new technologies (12). Conducting a readiness assessment is essential to understand the current environment and identify potential barriers to innovation, thereby minimizing the likelihood of implementation failure and avoiding unnecessary expenditure of resources (13). Health professionals’ readiness assessment is crucial for pre-implementation evaluation, which evaluates human factors through core and engagement readiness and determines individual skills readiness (14).

Success factors for electronic records implementation include individual readiness, organizational preparedness, and health professionals’ attitudes and skills. Challenges include high costs, inadequate funding, large populations, remote communities, limited technology availability, and lack of technical expertise, low computer skills, and insufficient facilities in developing countries, which hinder the adoption of EMRs (15–17).

Despite significant investments in EMRs, its successful implementation remains a global challenge (9, 18). The Ethiopian government has shown some interest in digital health initiatives, including EMRs, as part of its broader Health Sector Transformation Plan (HSTP), but the implementation has been slow due to competing priorities and limited funding (5, 17, 19). Numerous studies have documented low adoption rates and suboptimal outcomes of EMR systems, particularly in resource-limited settings like Ethiopia, and while previous studies have highlighted significant barriers such as limited infrastructure, technical expertise, financial constraints, and resistance among healthcare professionals, they have also emphasized potential benefits like improved patient care and data management (20–22). However, understanding the specific factors that influence readiness in this context is crucial, as they may vary across different settings due to technological and socio-demographic differences (23). The literature search found that more studies have focused on organisational readiness than on health professionals’ readiness, which is also important to ERMs implementation (6, 21), and no research has been conducted in the setting of our study area as per our search. Therefore, this study aimed to assess the factors influencing the readiness of healthcare professionals to adopt EMRs in public hospitals of Southern Oromia, Ethiopia.

## Methods and materials

### Study setting, design, and period

A facility-based cross-sectional study was conducted in public hospitals in the West Guji and Borena zones of Southern Oromia, Ethiopia, from May 1–30, 2024. There are a total of 9 public hospitals in these two zones, none of which had implemented EMRs at the time of the study. In the West Guji zone, there are four public hospitals: Bule Hora University Teaching Hospital, Kercha Primary Hospital, Melka Sodda Primary Hospital, and Gelana Primary Hospital. These hospitals have 285, 60, 74, and 52 health professionals, respectively. The total number of health professionals in West Guji public hospitals is 471. In the Borena Zone, there are five public hospitals: Yabelo General Hospital, Moyale, Mega, Teltele, and Arero Primary Hospitals. These hospitals have 163, 98, 65, 68, and 52 health professionals, respectively. Nursing is the dominant profession, on average making up 30%–50% of professionals while the gender ratio is predominantly male, with 1 in 4 health professionals being female in both zones` public hospitals. The total number of health professionals in Borena Zone public hospitals is 446.

### Population, sample size determination, sampling technique, and procedure

The study was conducted among all health professionals working in the public hospitals of these two zones and health professionals who have worked for less than six months were excluded in the study. The sample size was calculated using a single population proportion formula considering the assumptions: 95% level of confidence, a margin of error (*d*) of 5%, adding a 10% non-response rate, and the proportion of overall readiness of health professionals taken from the previous similar study conducted in public General Hospitals of Sidama region, southern Ethiopia “(*p*) is 36.5%” (24).

$$n=\frac{\left[Z\alpha /2^{2}\times p(1-p)\right]}{d^{2}}$$

$$n=\frac{{\left(1.96\right)}^{2}\times \;0.365(1-0.365)}{0.05^{2}}$$

n=356

**Figure 1:**
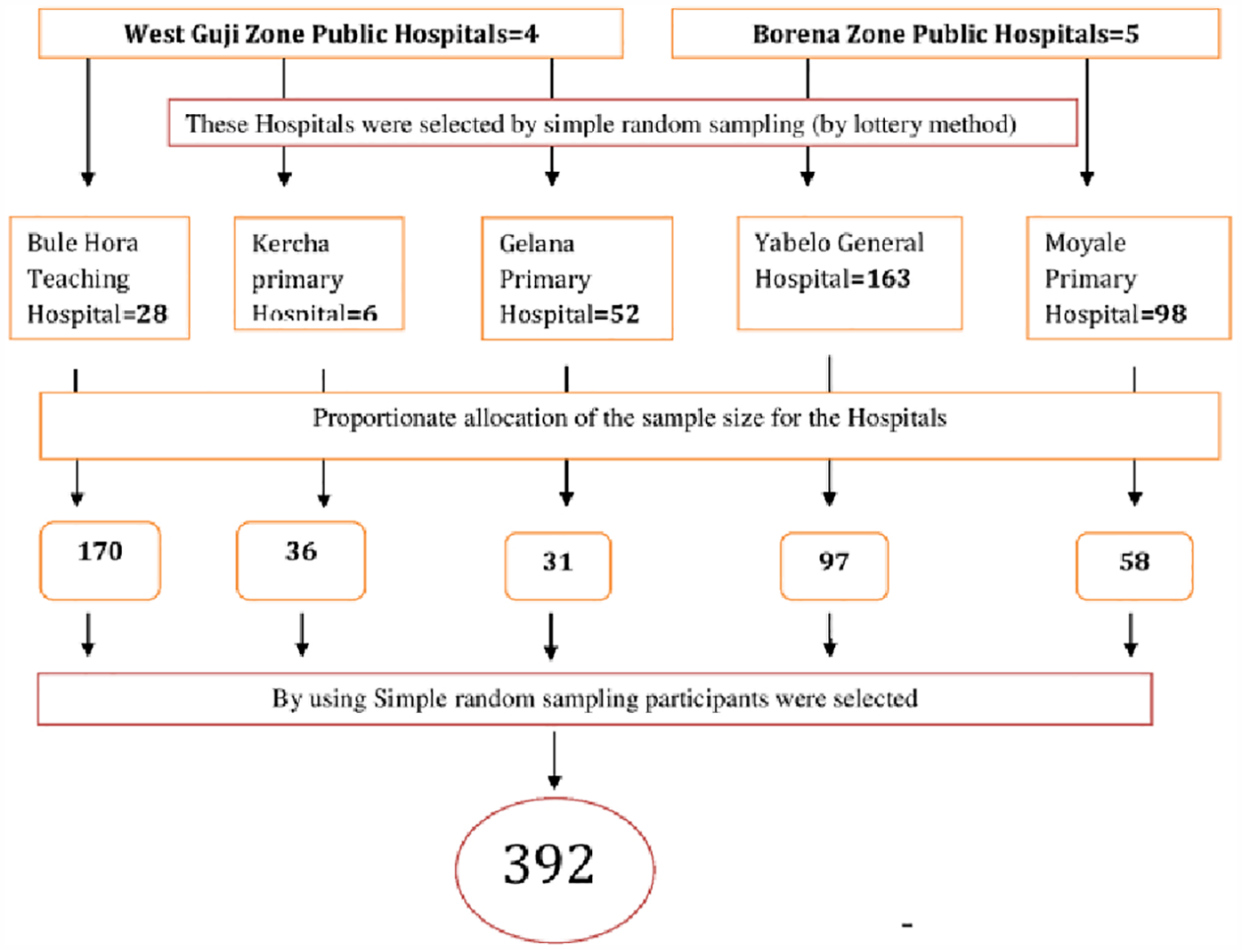
Schematic presentation of the sampling procedure among Health professionals working in West Guji and Borena Zones public hospitals, Oromia regional state, Southern Ethiopia, 2024.

Where *n* is the desired minimum sample size, *Zα*/2 is the standard normal deviate of 1.96, which corresponds to a 95% confidence level (*z* value at alpha = 0.05). The final required minimum sample size (*n*) after adding nonresponse is 392. Five public hospitals in West Guji and Borena Zones were selected using simple random sampling using the lottery method among nine hospitals in two zones. The reason for choosing these hospitals without EMR implementation is that the study aims to evaluate the readiness of health professionals, which is crucial for adopting an EMR system. The sample size was calculated proportionately based on the total number of professionals in each hospital, with inclusion and exclusion criteria applied. The quarterly report format of the hospital's human resources served as the sampling frame, with inclusion and exclusion criteria applied. Study subjects were selected and recruited through simple random sampling from each sampling frame (see Figure 1).

### Data collection tools, techniques, procedures, and quality management

The questionnaires were adapted from reviewed literature, and their relevance and reliability in assessing health professionals’ readiness have been validated in similar settings (2, 21, 23, 25, 26). The questionnaire contains six sections. The study used 73 questions to assess socio-demographic, technical, and organizational, knowledge about the EMR system, attitudes towards EMRs, and readiness for EMRs. Data were collected by six Health information technology professionals using a structured and pre-tested self-administered English version questionnaire. A pre-test was conducted among 20 participants at Gedeb Primary Hospital, which was not included in the study but had similar characteristics to the actual study participants. Necessary modifications were made after the pre-test before actual data collection, including the wording of specific questions to reduce ambiguity. Data collectors and supervisors received two days of training on data collection methods, participant approach, and ethical principles.

Questionnaires were reviewed and cross-checked daily and corrective discussions were held with all data collectors. Prior arrangements were made with hospital managers to visit the facilities at convenient times, and participants were allowed to complete the questionnaire at their convenience to avoid interrupting service delivery. Reliability was evaluated using Cronbach's alpha coefficient the values obtained were 0.85 for core readiness and 0.77 for engagement readiness.

### Study variables

#### Dependent variable

The primary outcome variable for this study was Health professionals’ readiness, which is the intersection of core readiness and engagement readiness.

#### Independent variables

Socio-demographic characteristics include age, sex, profession, education status, and duration of service. Technical factors such as computer literacy, computer skills, owning a personal computer, and organizational factors like computer availability at the office, computer training, internet access in the office, availability of IT support staff, and standby generator in the organization are also considered. Finally, knowledge of EMRs and attitude towards EMRs are independent variables in the study.

### Measurements of variables

#### Computer skills

were assessed using a five-point Likert scale to measure respondents’ proficiency in utilizing computers to use the ERM system's features. The scale ranged from strongly disagree (1) to strongly agree (5) and included items evaluating basic computer operations, such as entering, retrieving data, organizing, reporting, troubleshooting, and software applications. A median split was employed to categorize respondents into two groups: those with adequate computer skills, scoring at or above the median on the computer skills scale, and those with inadequate computer skills, scoring below the median (27).

#### Computer literacy

Refers to the knowledge and skills related to computers, including the ability to access, communicate, process, and comprehend basic information to make informed health decisions. This was assessed using a 5-point Likert scale ranging from “1 = strongly disagree” to “5 = strongly agree.” Respondents who scored at or above the median were classified as having adequate computer literacy, while those who scored below the median were classified as having inadequate computer literacy (23).

#### Knowledge about EMRs

To assess healthcare professionals’ knowledge of EMRs, a validated questionnaire comprising 11 items was administered. This instrument measured respondents’ understanding of basic EMR concepts and functionalities. A median split was employed to categorize participants into two groups: those with good knowledge [scoring at or above the median and those with poor knowledge, scoring below the median (26)].

#### Attitude towards EMRs

To assess participants’ attitudes towards EMRs, a validated 15-item Likert scale questionnaire was employed based on self-reported perceptions from healthcare professionals. Each item was rated on a five-point Likert scale ranging from “strongly disagree” to “strongly agree.” A total score was calculated for each participant, with higher scores indicating a more favourable attitude towards EMRs. A median split was used to categorize participants into two groups: those with a favourable attitude (scores at or above the median) and those with an unfavourable attitude, scores below the median (24).

#### Core readiness

The core readiness assessment results were determined by 13 questions measured on a four-point Likert scale. These questions addressed inefficient documentation of patient records, breached patient privacy, sharing of patient records, and healthcare providers’ dissatisfaction with completeness and accuracy. Health professionals who scored above or equal to the median were labelled as having core readiness (24).

#### Engagement readiness

This was measured by a set of 12 questions on a four-point Likert scale. These questions assess the active willingness and participation of people in the idea of EMRs, as well as the assessment of the risks, advantages, and disadvantages of EMRs. Health professional who scored above or equal to the median was labeled as having engagement readiness (24, 28).

**Overall readiness** is the intersection of core readiness and engagement readiness. Health professionals who possess both core readiness and engagement readiness are considered to have overall readiness (2, 24).

**Health professionals** include general practitioners, specialists of any type, optometrists, physiotherapists, medical radiologists, anesthetists, health officers, midwives, nurses, pharmacists, laboratory technicians (technologists), and health information technologists (29).

### Data processing and analysis

Data were checked for completeness and consistency, then entered into Epi-data version 4.6 and exported to SPSS version 27 for analysis. Descriptive statistics were computed to describe the different variables in the study. Bi-variable and multivariable binary logistic regression analyses were performed to determine the association of each independent variable with the outcome variable. Variables with a *p*-value <0.25 in the bi-variable analysis were entered into the multivariable logistic regression analysis. Diagnostics for multicollinearity indicated collinearity between variables, with variance inflation factor (VIF) values of for variables included in the multivariable being less than five, and the Hosmer & Lemeshow Goodness of Fit test was conducted to evaluate the appropriateness of the variables in predicting the dependent variables, resulting in a value of 0.75. The strength of the association was determined using the adjusted odds ratio with a 95% confidence interval, and a *p*-value less than 0.05 was considered statistically significant.

### Ethics approval and consent to participate

The study was conducted after obtaining ethical approval from Bule Hora University's Ethical Review Board with ethical approval number I/H/I/R/B/032/14. Permission was also sought from the administrative offices of the West Guji and Borena Zones, as well as the respective hospitals. All participants were provided with clear explanations of the study's objectives and potential benefits. Their written consent was obtained in exchange for their cooperation. Personal information that could potentially compromise the confidentiality of the respondents was not disclosed. The confidentiality and privacy of the participants’ information were maintained, and their decision to withdraw or not participate was respected.

## Results

### Socio-demographic characteristics

A total of 384 health professionals participated in the study, yielding a 98% response rate. The majority of participants were male (77.3%) and aged 29–35 years (54.4%), with a mean (±SD) age of 31.39 ± (4) years. Most participants held a Bachelor's degree (75%) and had over two years of work experience (67.4%). Notably, only 26% of participants possessed a personal computer (Table 1).

**Table 1:** Socio-demographic characteristics among health professionals at public hospitals in West Guji and Borena Zones, Southern Oromia, Ethiopia, 2024 (*n* = 384).

Variables	Category	Frequency	Percentage %
Sex of participants	Female	87	22.7
	Male	297	77.3
Age	21–28	102	26.6
	29–35	209	54.4
	>35	73	19.0
Profession of participants	Medical doctor	53	13.8
	Health Officer	3	0.8
	Nurse	180	46.9
	Midwifery	46	12.0
	Pharmacy	43	11.2
	Medical Laboratory	31	8.1
	Radiologist	6	1.6
	Anesthesia	5	1.3
	Optometrist	3	0.8
	Psychiatry	5	1.3
	Health Informatics	5	1.3
	Emergency Surgeon	4	1.0
Educational status of participants	Diploma	59	15.4
	Bachelor Degree	288	75.0
	Master's degree &above	37	9.6
Experience	≤1 year	41	10.7
	1–2 years	84	21.9
	>2 years	259	67.4
Have own Computer	Yes	100	26.0
	No	284	74.0

### Health professionals’ technical skills

**Figure 2:**
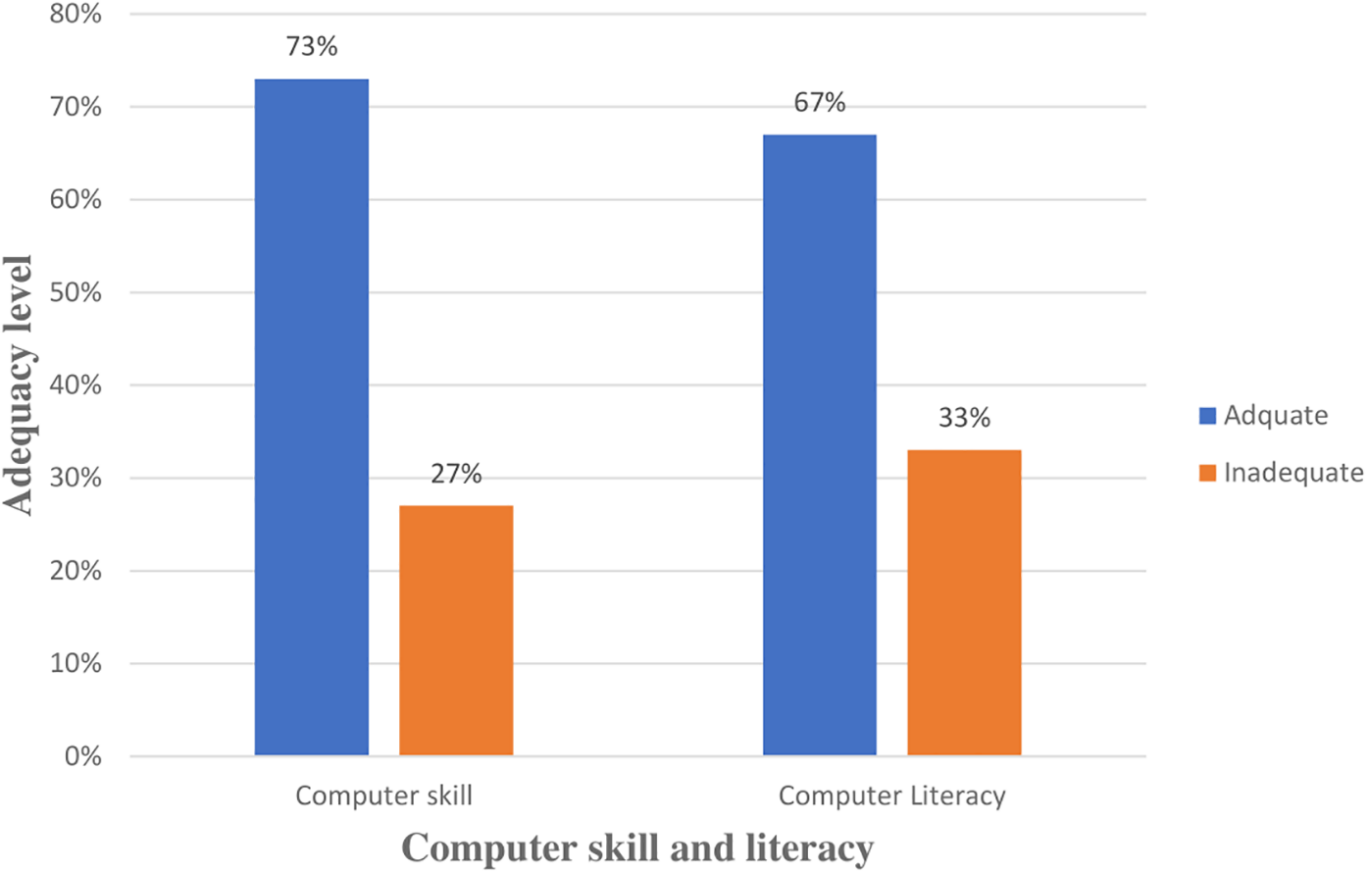
Health professionals’ technical related factors for assessing readiness at public hospitals of West Guji and Borena Zones, Southern Oromia, Ethiopia, 2024.

In this study, 280 (73%) health professionals had sufficient computer skills. Likewise 259 (67%) health professionals had high computer literacy (Figure 2).

### Organizational related characteristics

Of the 384 healthcare workers polled, 40.9% had access to a computer in their office, but just 2.3% had received formal computer training. Furthermore, only 3.6% had internet access at their workplace. Despite these constraints, a significant majority (96.1%) reported the presence of a standby generator at their respective institutions, indicating a level of infrastructural preparedness (Table 2).

**Table 2:** Organizational factors for assessing health professionals’ readiness at public hospitals of West Guji and Borena Zones, Southern Oromia, Ethiopia, 2024 (*n* = 384).

Variables	Category	Frequency	Percentage (%)
Computer availability at office	Yes	157	40.9
	No	227	59.1
Computer training	Yes	9	2.3
	No	375	97.7
Internet accessibility in office	Yes	14	3.6
	No	370	96.4
IT support staff availability	Yes	21	5.5
	No	363	94.5
Standby generator availability	Yes	369	96.1
	No	15	3.9

**Table 3:** Multivariable analysis of factors associated with health professionals’ readiness at public hospitals of West Guji and Borena Zones, Southern Oromia, Ethiopia, 2024 (*n* = 384).

Variables	Category	Readiness		COR (95%CI)	AOR (95%CI)
		Ready 232 (60.4%)	Not ready 152 (39.6%)		
Personal computer	Yes	83 (35.8%)	17 (11.2%)	4.42 (2.50,7.83)	3.54 (1.76,7.11)*
	No	149 (64.2%)	135 (88.8%)	1	1
Computer skill	Sufficient	194 (83.6%)	86 (56.6%)	3.92 (2.44,6.29)	2.49 (1.41,4.39)*
	Insufficient	38 (16.4%)	66 (43.4%)	1	1
Computer literacy	Adequate	187 (80.6%)	72 (47.4%)	4.617 (2.92,7.82)	2.67 (1.53, 4.66)*
	In adequate	45 (19.4%)	80 (52.6%)	1	1
Attitude towards EMRs	Favorable	171 (73.7%)	61 (40.1%)	4.18 (2.71,6.47)	2.77 (1.66, 4.63)*
	Unfavorable	61 (26.3%)	91 (59.9%)	1	1
Knowledge about EMRs	Good	172 (74.1%)	69 (45.4%)	3.45 (2.23,5.32)	2.56 (1.53, 4.29)*
	Poor(ref)	60 (25.9%)	83 (54.6%)	1	1
Age	21–28	70 (30.2%)	32 (21.1%)	1.701 (0.92,3.18)	2.66 (1.06,6.67)*
	29–35	121 (52.2%)	88 (57.9%	1.073 (0.63,1.83)	2.17 (1.05,4.51)*
	>35(ref)	41 (17.7%)	32 (21.1%)	1	1
Educational status	Diploma	42 (18.1%)	17 (11.2%)	1	1
	Degree	160 (69%)	128 (84.2%)	0.51 (0.28,0.93)	0.46 (0.19,1.12)
	Masters and above	30 (12.9%)	7 (4.6%)	1.74 (0.64,4.70)	0.97 (0.23,0.4.01)
Internet access	Yes	13 (5.6%)	1 (0.7%)	8.96 (1.16,69.25)	3.66 (0.401,33.36)
	No	151 (99.3%)	219 (94.4%)	1	1
Stand-by Generator availability	Yes	218 (59.1%)	151 (40.9%)	0.10 (0.013,0.793)	0.19 (0.023,1.606)
	No	14 (93.3%)	1(6.7%)	1	1

COR: Crude odd ratio, AOR: Adjusted odd ratio 1: reference categories, CI: Confidence interval.

* Indicates significance at 5% level.

### Health professionals’ knowledge about and attitude towards the EMR system

**Figure 3:**
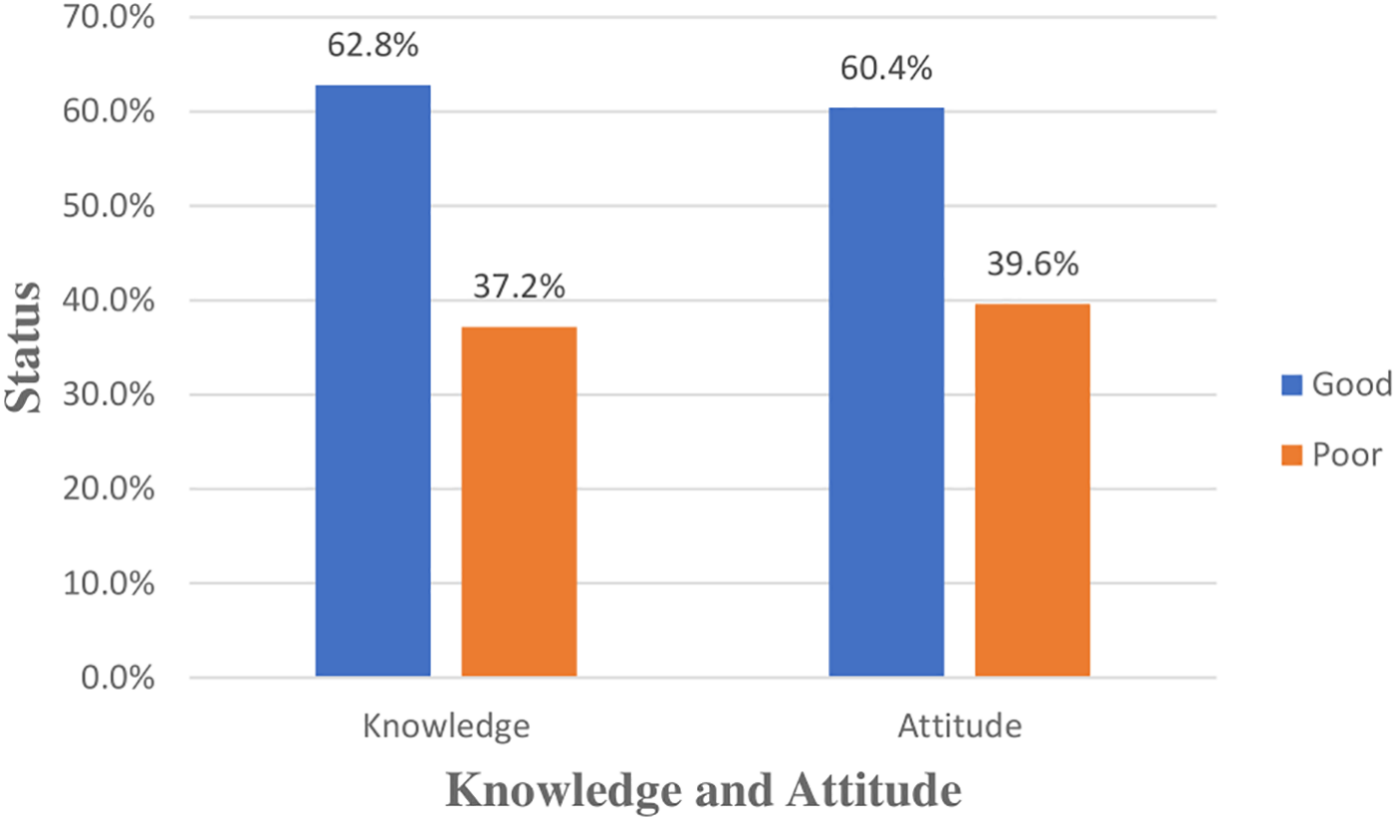
Health professionals’ knowledge about and attitude towards the EMR system at public hospitals in the West Guji and Borena Zones of Southern Oromia, Ethiopia, 2024.

In this study, the majority of health professionals, 62.8% [95% CI: (58.3%, 67.3%)], had good knowledge about EMRs. Similarly, 60.4% [95% CI: (56%, 64.8%)] of participants had a favorable attitude towards EMRs (Figure 3).

### Readiness of health professionals to implement the EMR system

**Figure 4:**
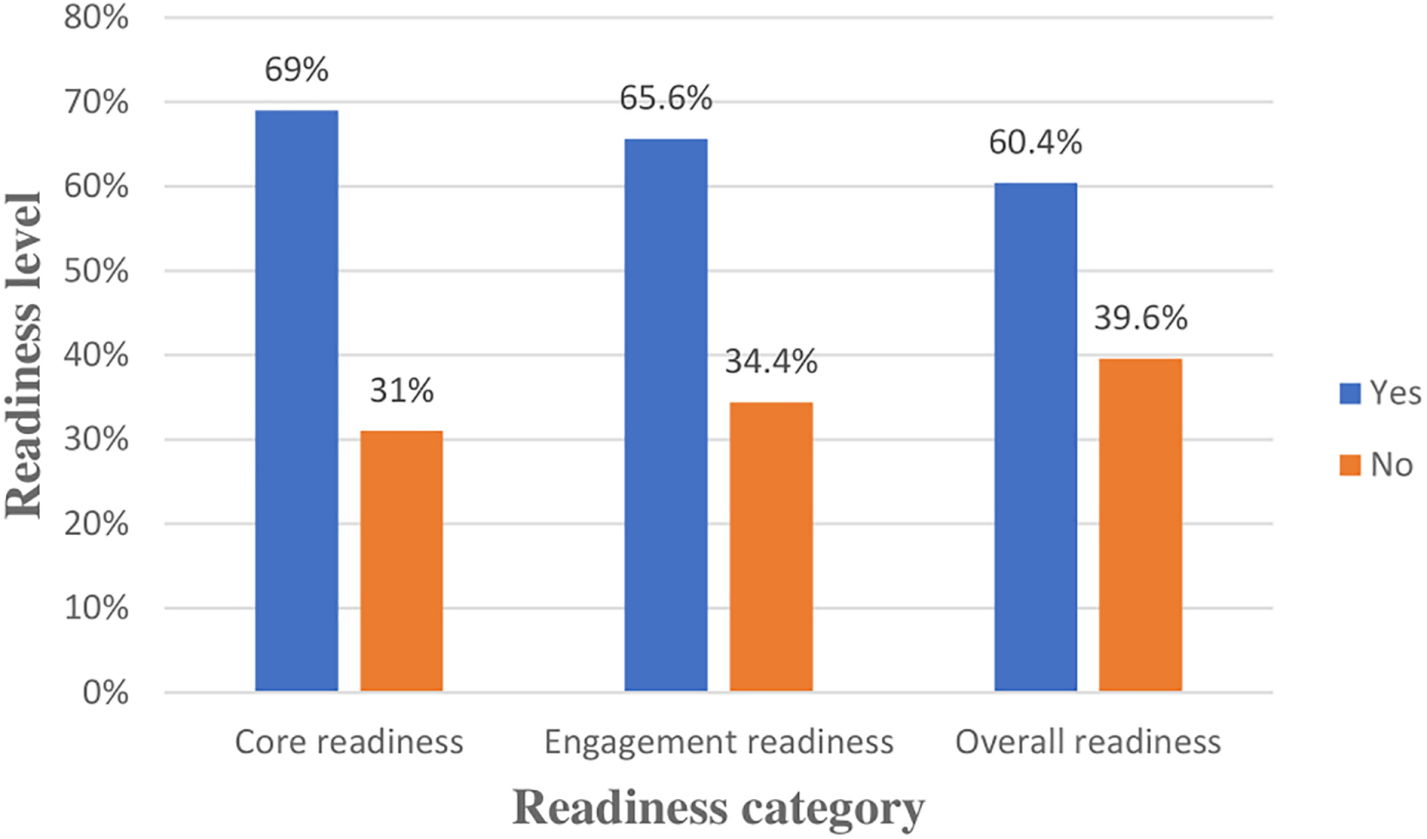
Health professionals’ readiness at public hospitals of West Guji and Borena Zones, Southern Oromia, Ethiopia, 2024.

Among all study participants, 60.4% [95% CI: (55.5%, 65.3%)] had overall readiness for an EMR system (Figure 4).

### Factors associated with health professionals’ readiness to implement EMR system

In this study age, educational status, ownership of a personal computer, computer skills, computer literacy, knowledge, attitude, internet accessibility at the office, and generator availability in the organization were variables that showed *p*-value <0.25 in bivariable analysis and became candidates for multivariable analysis.

After controlling for potential confounding variables through multivariable analysis, it was found that Health professionals aged between 22 and 28 were about 2.66 times more likely to be ready to adopt EMRs compared to those aged above 35[AOR = 2.66, 95% CI: (1.06, 6.67)] and respondents age between 29 and 35 were about 2.17 times more likely to be ready to adopt EMRs compared to those aged above 35 (AOR = 2.17, 95% CI: [1, 06, 4.51].

Participants who owned a personal computer were about 3.54 times more ready for an EMR system compared to those who did not own one [AOR = 3.54,95% CI:(1.76,7.11)].Sufficient computer skills were also associated with higher readiness for EMRs**.** Health professionals with sufficient computer skills were about 2.49 times more likely to be ready for an EMR system compared to those with insufficient computer skills [AOR = 2.49,95% CI: (1.41, 4.39)].

Health professionals with high computer literacy were about 2.67 times more ready for an EMR system compared to their counterparts [AOR =  2.67, 95% CI: (1.53, 4.66)]. Health professionals who have good knowledge of the Electronic Medical Record (EMR) system were about 2.56 times more likely to be ready for its implementation compared to those with poor knowledge [AOR = 2.56, 95% CI: (1.53, 4.29)].

The study also found that, Health professionals who had a favorable attitude towards EMRs were about 2.77 times more likely to be ready for EMRs implementation [AOR = 2.77, 95% CI:(1.66, 4.63)] (Table 3).

## Discussion

The overall readiness among health professionals was found to be 60.4% [95% CI: (55.5%, 65.3%)], with core readiness of 69% and engagement readiness of 65.6%. This finding was consistent with previous studies conducted in Ethiopia, where a readiness percentage of 65.25% was reported in the Gamo zone of southern Ethiopia, and a percentage of 62.3% was reported in the Gondar zone of northern Ethiopia, respectively (2, 6). The comparable results of these studies could be attributed to the shared national healthcare system,government policies, limited internet access, infrastructure limitations, and leading to similar baseline conditions impacting EMRs readiness (17, 30).

In contrast, overall readiness reported in this study was higher than studies conducted in Myanmar (54.2%) (28), Ghana (54.9%) (31), South west Ethiopia (52.8%), Northern Ethiopia (54.1%) (23), Northwest Ethiopia (42.1%) (25) and the Sidama region (36.5%) (24). The discrepancy may attributed to the increasing global engagement of healthcare workers with digital platforms might influence readiness levels and the timing of the studies could play a role the studies could also contribute to the varied results observed. The timing of the studies could play a role, as conducting the study during a period of increased awareness, improved diagnostics, or changes in healthcare policies might result in a higher prevalence compared to older studies.

The current study revealed that the overall readiness is much lower than the study conducted in China and the United States, where the EMR adoption system was 85.3%, and 96% respectively (32). The disparity may be due to infrastructure limitations, government policy, technology, and cultural resistance to adopt a digital system.

This study revealed that the Odds of health professionals’ EMRs implementation readiness is 2.66 times higher among those aged 21–28 and 29- 35 compared to those aged over 35 years respectively, to be prepared to use EMRs. This finding aligns with previous reports that younger age is associated with higher engagement in using technology and adopting EMRs (21, 26, 33). This implies that younger individuals are early adopters of e-health, which strengthens existing research findings on e-health adoption (34).

Participants who owned a personal computer were 3.54 times odds of readiness for an EMR system implementation compared to those who did not own it. This finding was consistent with a study conducted in Nigeria (33) and in previous study having computer at work place has significant association with EMR system implementation (25). This implies that readiness EMRs is closely linked to having the requisite computer proficiency.

The odds of readiness to be prepared for EMR implementation is 2.67 times higher among Health professionals who had adequate computer literacy were compared to their counterparts. This finding was consistent with a study conducted in different findings from Ethiopia (2, 26, 34) and Ghana (31). This implies that utilizing EMRs is closely linked to having the requisite computer proficiency. This underscores the critical role that computer proficiency plays in the successful implementation of EMR systems.

The present study revealed that health professionals who had good knowledge of the EMR system 2.56 times odds to be ready for EMR implementation compared to those with poor knowledge. This finding is consistent with studies conducted in Ethiopia (21, 23, 25). This study also is consistent with findings from study conducted in Australia and (35, 36). This may be due to the understanding of the importance of EMRs and existing knowledge may contribute to their motivation to use the system.

This study reveals that the odds of readiness for EMR implementation were 2.77 times higher among health professionals who had favourable attitude compared to their counter parts. This finding is consistent with studies conducted in Ethiopia, and Saudi Arabia (2, 37). This may be explained by the fact that having a positive perception of EMRs can influence the readiness of healthcare workers to incorporate the system into their daily activities.

In current study we found that educational status, internet access at the workplace, and standby generators did not show a statistically significant association with the readiness of health professionals for EMR adoption. However, a study conducted in Myanmar reported a statistically significant association between education and readiness for EMR adoption (28). The variance in these findings could be attributed to differences in the EMR implementation process and training provided for EMR adoption between Ethiopia and Myanmar. It is possible that in Myanmar, there is a stronger emphasis on formal education and specific training programs that enhance readiness for EMR adoption.

### Limitations of the study

This study primarily focused on assessing healthcare professionals’ readiness for EMRs implementation and did not incorporate qualitative findings to supplement its results. It also did not extensively explore other dimensions of readiness, such as technological, societal, and broader organizational factors, aside from a limited number of variables. Consequently, the findings may not comprehensively capture the factors influencing EMRs adoption in facilities where EMRs are already operational. Additionally, the reliance on self-reported assessments of computer skills and literacy introduced the potential for social desirability bias, as subjective questions were used to measure these competencies.

## Conclusion

The study findings indicate that insufficient readiness to utilize the EMRs, with readiness significantly associated with younger age, personal computer ownership, adequate computer skills, high computer literacy, knowledge of EMRs and a positive attitude toward EMRs. To ensure a successful transition to digital health, hospital leadership should prioritize targeted training programs to enhance computer literacy and address knowledge gaps, particularly among older staff. Financial strategies, such as subsidies for personal computer ownership or partnerships with technology providers, could further support digital health initiatives. Additionally, implementing pilot programs or phased approaches to EMRs adoption would allow for gradual adaptation, minimize disruptions, and provide valuable insights for scaling up. Combined with strong leadership commitment, these measures can foster a smoother transition to EMRs and improve overall readiness among health professionals. Also, the adoption of EMRs requires institutional, policy, and societal efforts. Investments in digital infrastructure, regulatory frameworks, hospital leadership empowerment, public-private partnerships, and patient engagement can overcome barriers, promoting efficient, equitable, and patient-centered healthcare.

## Data Availability

The original contributions presented in the study are included in the article/Supplementary Material, further inquiries can be directed to the corresponding author.
