# Supplementary material for: Health professionals’ readiness for and factors influencing electronic medical record systems implementation in Southern Oromia, Ethiopia, 2024: a cross-sectional study
Source: Front Digit Health. 2025 Apr 10;7:1531315. doi: 10.3389/fdgth.2025.1531315 (PMC12018405; doi:10.3389/fdgth.2025.1531315)
Supplement: Supplementary file 1 [file Datasheet1.pdf]

Human Participants Research Checklist

Complete the following if your study involved human participants or human participants' data. These questions should be addressed for prospective and retrospective studies.

1. Did you obtain ethics approval for this study?
   - If yes, please upload (file type "Other") the original approval document you received from your ethics committee. If the original document is in another language, please also provide an English translation.

     ___ Uploaded ___ N/A

   - If you did not obtain ethical approval, please explain why this was not required below.

Yes With Ref. No/I/O/H/I/R/B/032/14

2. If you prospectively recruited human participants for the study – for example, you conducted a clinical trial, distributed questionnaires, or obtained tissues, data or samples for the purposes of this study, please report in the Methods:
   i. The day, month and year of the **start and end** of the recruitment period for this study.

   A cross-sectional study was conducted among 384 health professionals from May 1-30, 2024,

   ii. Whether participants provided informed consent, and if so, what type was obtained (for instance, written or verbal, and if verbal, how it was documented and witnessed). If your study included minors, state whether you obtained consent from parents or guardians. If the need for consent was waived by the ethics committee, please include this information.

The study was conducted after obtaining ethical approval from Bule Hora University's Ethical Review Board. Permission was also sought from the administrative offices of the West Guji and Borena Zones, as well as the respective hospitals. All participants were provided with clear explanations of the study's objectives and potential benefits. Their written consent was obtained in exchange for their cooperation. Personal information that could potentially compromise the confidentiality of the respondents was not disclosed. The confidentiality and privacy of the participants' information were maintained, and their decision to withdraw or not participate was respected

Completed ___ N/A

3. If you are reporting a retrospective study of medical records or archived samples, please report in the Methods section:
   i. the day, month and year when the data were accessed for research purposes
   ii. whether authors had access to information that could identify individual participants during or after data collection

___ Completed ___ N/A
